# Cell-derived extracellular matrix-coated silk fibroin scaffold for cardiogenesis of brown adipose stem cells through modulation of TGF-β pathway

**DOI:** 10.1093/rb/rbaa011

**Published:** 2020-04-24

**Authors:** Wei Liu, Yanfeng Sun, Xiaohui Dong, Qi Yin, Huimin Zhu, Siwei Li, Jin Zhou, Changyong Wang

**Affiliations:** r1 School of Life Science and Technology, Harbin Institute of Technology, 92 West Dazhi Street, Nan Gang District, Harbin 150080, P.R. China; r2 Tissue Engineering Research Center, Academy of Military Medical Sciences, 27 Taiping Road, Beijing 100850, P.R. China; r3 Department of Neural Engineering and Biological Interdisciplinary Studies, Institute of Military Cognition and Brain Sciences, Academy of Military Medical Sciences, 27 Taiping Road, Beijing 100850, P.R. China; r4 Pediatric, Clinical Cancer, The Armed Police General Hospital, 69 Yongding Road, Beijing 100080, P.R. China

**Keywords:** cell-derived extracellular matrix, cardiac fibroblasts, brown adipose stem cells, silk fibroin scaffolds

## Abstract

The cell-derived extracellular matrix (ECM)-modified scaffolds have advantages of mimic tissue specificity and been thought to better mimic the native cellular microenvironment *in vitro*. ECM derived from cardiac fibroblasts (CFs) are considered as key elements that provide a natural cell growth microenvironment and change the fate of cardiomyocytes (CMs). Here, a new hybrid scaffold is designed based on silk fibroin (SF) scaffold and CFs-derived ECM. CFs were seeded on the SF scaffold for 10 days culturing and decellularized to produce CFs-derived ECM-coated SF scaffold. The results showed that the cell-derived ECM-modified silk fibroin scaffold material contained collagen, laminin, fibronectin and other ECM components with myocardial-like properties. Further to explore its effects on brown adipose stem cells (BASCs) differentiation into CMs. We found that the CF-derived ECM-coated scaffold also increased the expression of CM-specific proteins (e.g. cardiac troponin T and α-actinin) of BASCs. Notably, the β1-integrin-dependent transforming growth factor-β1 signaling pathway was also involved in the regulation of CF-derived ECM by promoting the differentiation of BASCs into CMs. Overall, these findings provide insights into the bionic manufacturing of engineered cardiac tissues (ECTs) and establish a theoretical basis for the construction of ECTs.

## Introduction

Cardial tissue engineering has developed rapidly in recent years, and engineered cardiac tissues (ECTs) have been constructed *in vitro* based on different scaffold guided by various construction strategies [1–3]. Scaffold as a part of myocardial tissue engineering play important roles in myocardial remodeling. In recent years, more and more researchers have begun to pay attention to modified scaffold; for example, carbon nanotube material composite gelatin has conductive properties [4], and fullerenol nanoparticle-modified chitosan scaffolds have antioxidant and conductive properties [5]. These materials can promote disk formation and enhance the electrical integration between ECTs and the host myocardium. Studies have shown that the components, stereo structure, hardness and microenvironment-regulating ability of the scaffold material can affect cardiomyocyte (CM) remodeling and the quality of the reconstructed ECTs [6, 7]. Accordingly, the choice and functionalization of the material are important considerations when constructing ECTs. 

Functionally scaffolds modified with cell-derived extracellular matrix (ECM) have been developed; some studies also used ECM derived from terminally differentiated cells, stem cells to explore the developmental rules of stem cell osteogenesis, adipogenesis and differentiation into nerve cells [8–11]. Gu *et al.* [12] used Schwann cell-derived ECM-modified chitosan/silk fibroin (SF) for bridging rat sciatic nerve gaps. Cardiac fibroblasts (CFs) are important stromal cells that secrete ECM components, such as collagen, in the heart. CFs account for ∼60–70% of the total number of normal myocardial tissue cells, which are widely present in the heart and surround CMs [13, 14]. ECM secreted by CFs is also an important component of the cardiac matrix and is closely related to cardiac development, structure, cellular signaling systems and electromechanical function [15, 16]. Therefore, CF-derived matrix materials have attracted much attention because of the presence of micro environmental factors that promote cell growth. One report used a CF-derived matrix to promote the maturation of neonatal rat CMs [17]. However, no studies have described the use of a CF-derived matrix for modification of tissue engineering scaffolds.

SF scaffold is a kind of traditional scaffold materials that show good bioactivity and biocompatibility, which may facilitate the construction of tissues, such as tendons, cortex and bones [18–20]. However, the functionality of SF scaffold still needs to be further improved. Decellularized ECM is a biomaterial that best mimics the native cellular microenvironment, showing good bioactivity, biodegradability and biocompatibility. The ECM acts as a cellular support and directs cell fate through coordinated physical and biochemical cues. Thus, ECM may have applications in the modification of SF scaffold for material modification and myocardial remodeling.

In this study, we aimed to construct a CFs-derived ECM-coated SF scaffold and further inoculate BASCs to explore its differentiation and related mechanisms. The silk protein scaffold is considered to be a natural biocompatible material, and the ECM derived from CFs can effectively mimic the myocardial microenvironment. At the same time, Q-PCR, western blotting, immunofluorescence and other methods were used to clarify the rule of proliferation and differentiation into CMs. And further revealed the regulation of brown fatty stem cells differentiation into CMs by β1-integrin-dependent TGF-β1 signaling pathway. The research provides a reference for the construction of biomimetic scaffolds combined with cell-derived ECM for constructing ECTs.

## Materials and methods

### Isolation and cultivation of rat CFs and BASCs

CFs were isolated as reported previously [21]. Briefly, hearts were obtained from 1-day-old Sprague-Dawley rats and cut into pieces. The tissue pieces were then placed in 5 ml of 0.05% trypsin and digested at 37°C for 5 min repeatedly until the tissue disappeared. The supernatant was collected and centrifuged at 1200 g for 7 min to obtain cell pellets, and collected cells were resuspended in Dulbecco’s modified Eagle’s medium (DMEM) supplemented with 10% (v/v) fetal bovine serum (FBS) for 1 h to enrich for CFs by allowing attachment of fibroblasts. The culture medium was changed every other day for subsequent analysis.

As reported previously, BASCs were isolated from the interscapular adipose tissue of male Sprague-Dawley rats [12]. Briefly, the interscapular adipose tissue was isolated and washed with phosphate-buffered saline (PBS), chopped up with scissors. The tissues were digested by digest solution which containing with 0.1% collagenase IV (Sigma), 0.1% dispase II (Roche) and 0.05% trypsin (Gibco) for 45 min at 37°C. The digested cells were cultured in α-MEM (Invitrogen) containing 15% FBS (Invitrogen) for cell culture on culture dishes or CF-derived ECM-modified biomaterial (10 × 10 mm).

### ECM derived from cultured CFs

The cultured CFs are digested and inoculated in 24-well plates, and then decellularized after 10 days of culture. Cells were incubated in warm 0.5% Triton X-100 solution in PBS supplemented with 20 mM ammonium hydroxide (Sigma) for 5 min at 37°C to obtain the decellularization ECM.

### Fabrication of ECM-modified silk scaffold

The formation of ECM-modified silk scaffold involves three steps. First, SFs were prepared from *Bombyx mori* cocoons as described previously [22]. Briefly, silk cocoons were boiled in Na_2_CO_3_, and then dissolved in 9.3 M LiBr solution and dialyzed. The final concentration of the SF aqueous solution was 8% (w/v). The porous SF scaffold was further prepared by the method of sinking salt. Second, the cultured CFs were seeded at 1 × 10^6^, and the CFs on the scaffold were cultured for 10 days, accompanied by adhering, growing and secreteing a large amount of ECM components. Third, the CFs on the SFs cultured for 10 days were decellularized. The method is as follows: the SFs were incubated in warm 0.5% Triton X-100 solution in PBS supplemented with 20 mM ammonium hydroxide (Sigma) for 5 min at 37°C to obtain the decellularization ECM. Finally, ECM-modified silk scaffold was obtained and soaked in serum-free DMEM medium for later use.

### Transmission electron microscopy

Cell-derived ECM samples were dried in a freeze dryer (SCIENTZ-10N) overnight, coated with gold particles and then visualized using a transmission electron microscope (Technai10; Philip).

### Total protein assay

After decellularization, cell-derived ECM samples were quantified by detecting total protein using a BCATM Protein Assay Kit (Thermo Scientific). The amount of collagen in decellularized ECM was measured using Sirius Red Staining.

### Total DNA assay

After decellularization, removed cell samples were quantified by measuring DNA concentrations with a TIANamp Genomic DNA kit (Tiangen) as described previously. Native CF-derived ECM and ECM-modified scaffold were lysis using 200 μl cell lysis buffer, centrifuged at 1000 g for 1 min, and treated with 20 μl proteinase K. Adding 200 μl buffer GB to treate the homogenates at 70°C for 10 min. Next, 200 μl absolute ethanol was added, and the samples were shaken for 15 s; the resulting solution was centrifuged at 12 000 g for 30 s. The samples were added to 500 μl buffer GD and centrifuged at 12 000 rpm for 30 s. The sample homogenates were added to 600 μl buffer PW, transferred to filter spin columns and centrifuged at 12 000 rpm for 30 s. Finally, the extracted DNA was eluted from column filters into sterile 1.5-ml Eppendorf tubes and centrifuged at 12 000 rpm for 2 min. The concentrations of double-stranded DNA in the samples were quantified by measuring the fluorescence intensity using a microspectrophotometer (AVans).

### Immunochemical staining

ECM samples and ECM-modified scaffolds were fixed with 4% paraformaldehyde, after washing with PBS, the samples were treated with 0.3% triton and then 5% goat serum blocked, and immunostained with mouse polyclonal antibodies against fibronectin (1:200; Abcam) and rabbit polyclonal antibodies against laminin, tubulin, collagen I, collagen III, collagen IV and F-actin (1:200; Abcam) overnight at 4°C. Subsequently, the samples were treated for 2 h with different fluorescein isothiocyanate (FITC)-conjugated or Cy3-labeled secondary antibodies (Boster) at 37°C for 2 h the next day. The samples were then visualized using a confocal laser-scanning microscope (Nikon A1).

### Interaction between ECM-modified silk scaffold and BASCs

Primary isolated BASCs (2 × 10^6^ cells) were seeded onto the ECM-modified silk scaffold. No ECM silk scaffold was used in the control. The samples were cultivated in α-MEM medium containing 15% FBS for 14 days. The proliferation and morphology of BASCs on the SF scaffold and ECM-modified scaffold were then evaluated.

### Live/dead staining

To assess cell viability by live/dead staining, cell-loaded SF scaffolds and ECM-modified scaffolds were incubated with 0.2 ml live/dead staining solution (Invitrogen) for 30 min [23]. After washing with PBS, the samples were examined under a confocal scanning microscope (Nikon A1) to distinguish live and dead cells.

### DNA content assay

To detect the cell proliferation seeded in scaffolds, BASCs were cultured as described above. Cells seeded in SF scaffolds without ECM served as a control. At different time points (days 1, 3, 5, 7, 9, 11 and 13), the cell-loaded SF scaffolds and ECM-modified scaffolds were harvested and frozen at −80°C. DNA contents were then measured using a TIANamp Genomic DNA kit (Tiangen) as described above, and proliferation curves were drawn using originPro.

### Immunofluorescence detection

ECTs were fixed with 4% paraformaldehyde, 0.1% Triton X-100 solution and 2% bovine serum albumin solution (for blocking) sequentially. Tissues were then incubated with primary anti-F-actin (1:200), anti-α-actinin (1:150) and anti-connexin (Cx) 43 (1:200) antibodies (Abcam) overnight at 4°C followed by incubation with appropriate FITC- or CY3-conjugated anti-rabbit secondary antibodies (1:100; Sigma). Staining cell nuclei by 4′,6-diamidino-2-phenylindole, and samples were visualized using a confocal microscope (Nikon A1).

### Gene expression detection

To detect the differentiation of BASCs into CMs on the scaffold, total cell RNA was extracted on days 3, 7 and 14 using TRIzol (Invitrogen). cDNA was synthesized using a high-capacity RT-PCR kit, and real-time PCR was conducted utilized with a SYBR Premix Ex Taq II Kit (Toyobo) to detect the transcript levels of myocyte enhancer factor 2C (*Mef2C*), NK2 homeobox 5 (*Nkx2.5*), ISL LIM homeobox 1 (*Isl1*), GATA-binding protein 4 (*Gata4*), cardiac troponin T (*cTnT*), α-actinin and glyceraldehyde 3-phosphate dehydrogenase (*GAPDH*; internal control). Primer sequences for real-time PCR are listed in Supplementary Table S1. The data were analyzed using real-time PCR system software (SynergyTM H1; BioTek Instruments, USA). The relative expression levels were normalized according to the Ct value of rat *GAPDH* (2^△CT^ formula).

### Western blot analysis

Cells in ECTs at 3 and 14 days were collected and lysed in ice-cold RIPA buffer. Harvest cell lysate through centrifugation at a rate of 12 000 g for 30 min. After quantification using a BCA Protein Assay Kit (Thermo Scientific), the proteins were separated by sodium dodecyl sulfate polyacrylamide gel electrophoresis on 10% gels and transferred to polyvinylidene fluoride membranes (Roche). The membranes were blocked in 5% defatted milk for 1 h at room temperature and then incubated with primary antibodies (Supplementary Table S2) at 4°C overnight. Membranes were washed with TTBS and then incubated with secondary antibodies (Cell Signaling Technology) at room temperature. Protein bands were detected by chemiluminescence reagent (Applygen). The band intensity was analyzed by Image J and normalized by GAPDH.

## Results

### Characterization of CF-derived ECM

**Figure 1:**
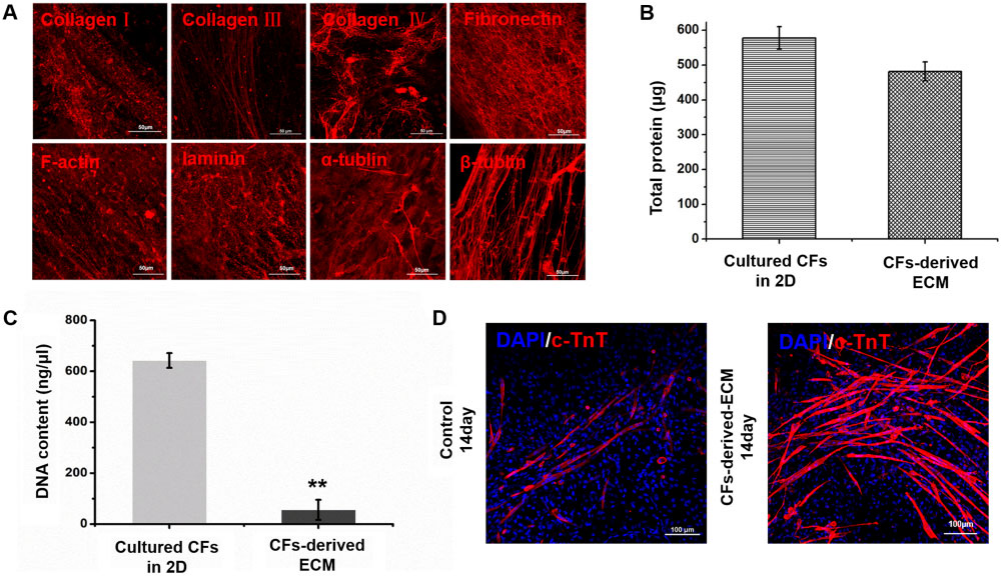
(**A**) Immunochemistry micrographs showing that collagen I, collagen III, collagen IV, fibronectin, F-actin, laminin, α-tublin and β-tublin were expressed in the CFs-derived ECM. Scale bar, 50 nm. (**B**) Total protein was detected between cultured CFs in 2D and CFs-derived ECM. (**C**) Histogram comparing the DNA content between cultured CFs in 2D and CFs-derived ECM (**P < 0.01). (**D**) Immunocytochemistry staining of cTnT shows the differentiation into CMs of BASCs cultured in no ECM group and CFs-derived ECM-coated group. Scale bar, 100 μm

CFs were continuously cultured for 10 days to form specific ECM components and structures, and the ECM was then obtained by decellularization. The ECM fraction was observed by immunofluorescence staining, and the results showed that CF-derived ECM contained collagens I, III and IV, although the content of collagen III was relatively low. We also detected large amount of fibronectin, F-actin, laminin, α-tubulin and β-tubulin in the cell-derived ECM. Fibronectin contents were increased, β-tubulin showed a directional filamentous arrangement and immunofluorescence staining indicated that the CF-derived ECM contained substances exhibiting biological activity (Fig. 1A). Protein content determination showed that most of the protein was left after decellularization (Fig. 1B). The results of DNA quantification indicated that most of the cellular DNA was removed, and the amount of remaining DNA was approximately 1% of the total DNA in the cells (Fig. 1C). In addition, BASCs were cultured on CF-derived ECM for 14 days, and immunofluorescence staining was used to detect the differentiation of BASCs into CMs. The results showed that BASCs cultured on the CF-derived ECM could differentiate into CM-like cells expressing the CM marker cTnT (Fig. 1D).

### Scaffold characterization

**Figure 2:**
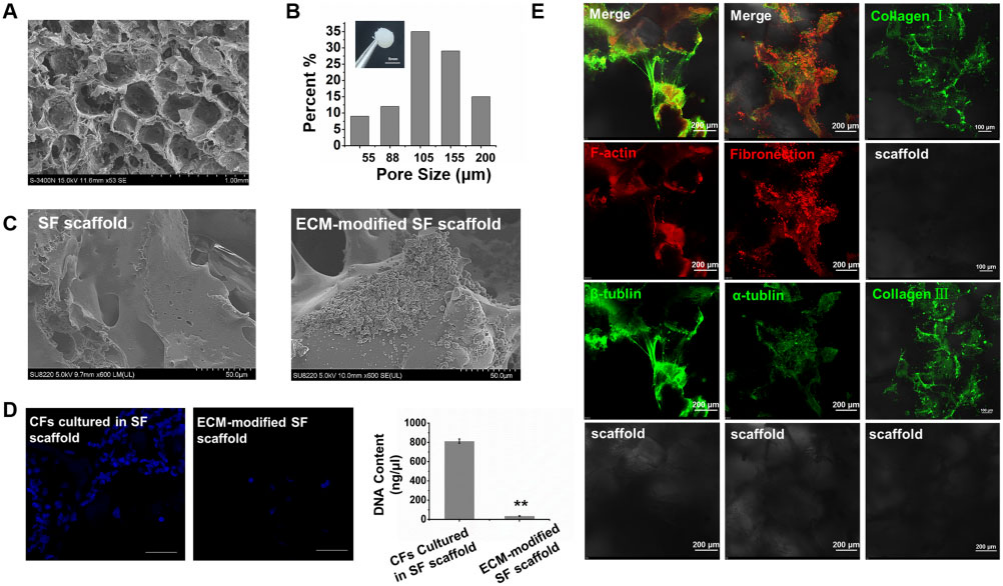
(**A**) Scanning electron micrographs of SF scaffold. (**B**) General picture of the SF and histogram showed the pore size of SFs. (**C**) SF and ECM-modified SF scaffold was detected by SEM. (**D**) DAPI staining and DNA content determination for nuclear residue on scaffold material after decellularization. The group of CFs cultured in SF scaffolds showed the distribution of the nuclei of CFs before decellularization. The group of ECM-modified SFs scaffold showed the few nuclei after decellularization. (**P < 0.01). (**E**) Immunochemistry showing that collagen I, collagen III, fibronectin, F-actin, α-tublin and β-tublin were expressed in the ECM-modified SF scaffold. Scale bar, 200 μm

First, we used scanning electron microscopy (SEM) to evaluate the SF scaffold. The results showed that the prepared SF scaffolds were relatively uniform in pore size (Fig. 2A), with most measuring 100–155 μm (Fig. 2B). Additionally, CFs could produce ECM, and CF-derived ECM could promote the differentiation of BASCs into CMs. We inoculated CFs on SF scaffolds for 10 days. Immunofluorescence showed that CFs on scaffolds expressed vimintin and evenly spread on the scaffold (Supplementary Fig. S1). CFs were cultured on the scaffold for 10 days and decellularized; subsequently, dense granulated matrix residues on the ECM-modified SF scaffold were observed by scanning electron microscopy (Fig. 2C). Further analyses showed that there were few residual nuclei after decellularization compared with that in nondecellularized cells. Measurement of DNA contents showed that most of the cellular DNA was removed during the decellularization process, the total DNA rarely remains after decellularization (Fig. 2D). Detection of ECM residues on SF scaffolds by immunofluorescence showed that after decellularization, the SF scaffold was modified by CF-derived ECM, including collagen I and III, fibronection, F-actin, α-tubulin and β-tubulin. Moreover, β-tubulin was filamentously connected between the pores of the SF scaffold (Fig. 2E).

### Effects of ECM-modified scaffold on survival of BASCs

The primary isolated BASCs were characterized by ﬂuorescence-activated cell sorting analysis (Supplementary Fig. S2). BASCs were positive for CD90, CD133 and CD29, but were negative for CD45, and CD34, which are endothelial cells and hematopoietic markers, respectively. Importantly, CD133- and CD90-positive cells have been reported to exhibit cardiogenic capacity [24].

**Figure 3:**
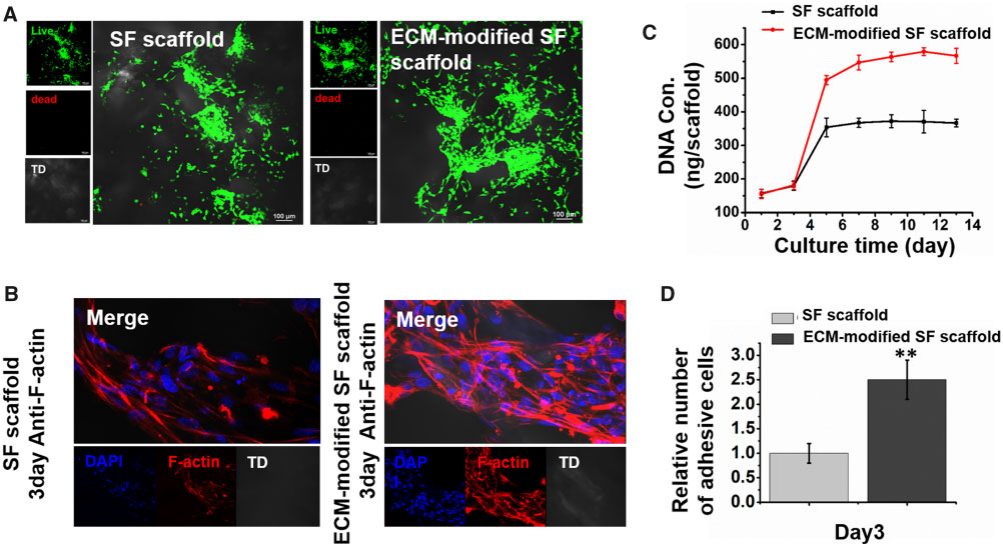
(**A**) Live/dead detected the cell viability of BASCs on SFs scaffold and ECM-modified SF scaffold. (**B**) Immunocytochemistry with anti-F-actin showing the cell adhesion of BASCs on SFs scaffold and ECM-modified SF scaffold in 3 days of culturing. Scale bar, 100 μm. (**C**) Determination of DNA content of BASCs on SF scaffold and ECM-modified SF scaffold cultured in different days (1/3/5/7/9/11/13 days). (**D**) Histogram comparing the number of cell adhesion between SFs and ECM-modified SFs scaffold group (**P < 0.01)

Primary isolated BASCs were inoculated onto SF scaffolds and ECM-modified SF scaffolds to construct ECTs. After 3 days of culture, live/dead assays showed that BASCs had good cell viability on the SF and ECM-modified scaffolds, without resulting in cell death (Fig. 3A). To further evaluate the effects of ECM-modified SF scaffolds on BASCs adhesion, immunofluorescence staining of F-actin was detected. The results showed that the morphology of BASCs on ECM-modified SF scaffolds became more stretched and formed thicker actin filaments compared with cells on SF scaffolds alone after 3 days of culture (Fig. 3B). To evaluate the correlations between ECM and BASCs cellular adhesion, we analyzed the cell number and area per field using Image J. The results revealed that ECM-modified scaffolds significantly enhanced the adherent area and the number of BASCs compared with BASCs on SF scaffolds (Fig. 3D).

To assess whether the ECM-modified SF scaffold affected the proliferation of BASCs, we collected ECT samples on different days and further evaluated the proliferation of BASCs on the scaffolds by measuring DNA contents. The results showed that compared with the SF scaffold alone, the ECM-modified scaffold significantly promoted the proliferation of BASCs, and the cells proliferated rapidly in 3–5 days, reaching a plateau on day 5. Overall, these studies confirmed that the ECM-modified SF scaffold promoted the adhesion, activity and proliferation of BASCs during the construction of ECTs.

### Cardiomyogenic expression

**Figure 4:**
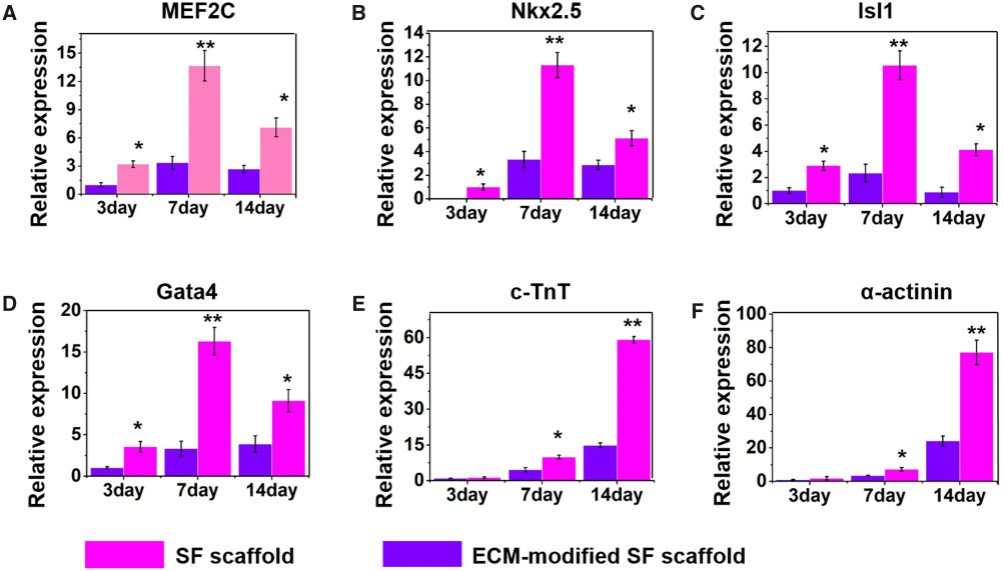
The expression of cardiac relative gene of BASCs cultured on SFs scaffold and ECM-modified SFs scaffold for different culturing day, including Mef2C (**A**), Nkx2.5 (**B**), Isl1 (**C**), gata-4 (**D**), c-TnT (**E**) and α-actinin (**F**) (*P < 0.05, **P < 0.01)

In order to further elucidate the effects of the ECM-modified SF on the cardiogenesis of BASCs, we evaluated the spontaneous differentiation of BASCs into CMs on the ECM-modified SF scaffold. Notably, the expression of *Nkx2.5*, *GATA4*, *Mef2c*, *Isl1*, α-actinin and *cTnT* was enhanced in BASCs on the ECM-modified SF scaffolds comparing with the cells seeding on SF scaffold alone. The expression level of *Nkx2.5*, *GATA4*, *Mef2C* and *Isl1* expressed in stem cells were significantly enhanced in BASCs on the ECM-modified SF scaffolds (Fig. 4A–D). However, the expression levels of these transcription factors decreased over time. When the cells were cultured for 3 days, the presence of ECM on the SF scaffolds significantly induced the expression of α-actinin and *cTnT*. In contrast, *Nkx2.5*, *GATA4* and *Mef2c*, which are early markers of CMs, showed high expression on day 7 in the SF group, whereas α-actinin and *cTnT*, which were markers of mature CMs, showed increased expression from 3  to 14 days of culturing (Fig. 4E and F). These data further implied that the ECM was an important factor to effect the differentiation of BASCs into CMs.

### ECM-modified scaffold-accelerated cardiogenesis in BASCs

**Figure 5:**
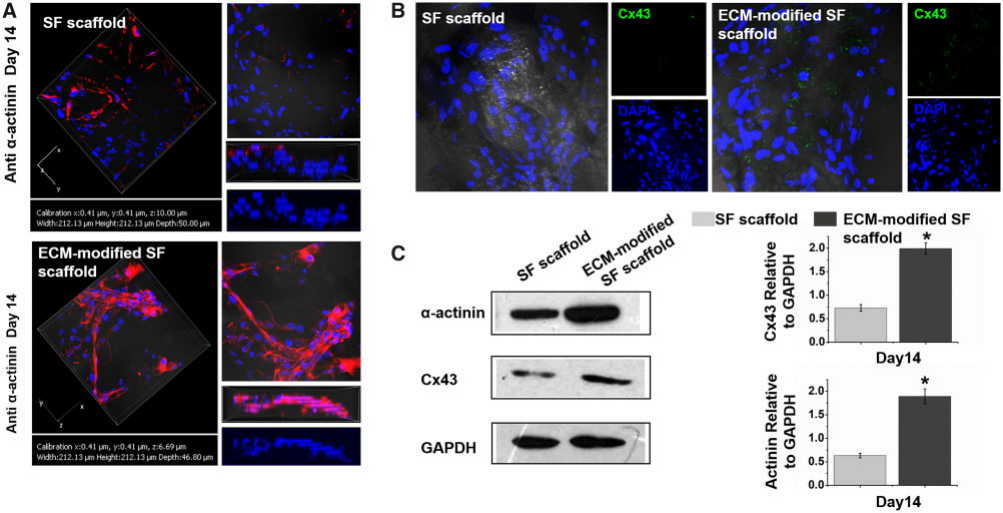
(**A**) Immunofluorescence staining of α-actinin was detected to evaluate the differentiation of BASCs seeded on the SF scaffold and ECM-modified SF group at day 14. (**B**) Immunofluorescence staining of cx43 was detected at day 14. (**C**) The expression of α-actinin and cx43 were detected by western blotting, they were significantly increased at day 14 for ECM-modified SF scaffold group by quantification analysis using image J. *P < 0.05

Based on the differentiation of BASCs in ECTs, we further evaluated the quality of ECTs cultured for 14 days. The results showed that BASCs expressed large amount of mature myocardium sarcomeric structure on ECM-modified SF scaffolds compared with that in cells cultured on SF scaffolds. Three-dimensional reconstruction of the constructed ECTs showed that the differentiated BASCs were distributed more in the scaffold material (Fig. 5A). Additionally, the expression of the connexin Cx43 was increased in BASCs found in ECTs constructed using the ECM-modified SF scaffold, whereas BASCs in the SF group showed almost no Cx43 expression (Fig. 5B). To further verify the expression of cTnT and Cx43 proteins, we used western blotting. The results showed that BASCs in ECTs constructed with the ECM-modified SF scaffold showed higher expression of cTnT and Cx43 compared with the control group on day 14 of culture, suggesting that the sarcomere assembly of CMs in ECTs and the ECTs formed using the ECM-modified scaffold were more mature (Fig. 5C).

### The pivotal role of transforming growth factor-β1 signaling pathway in cardiogenesis

**Figure 6:**
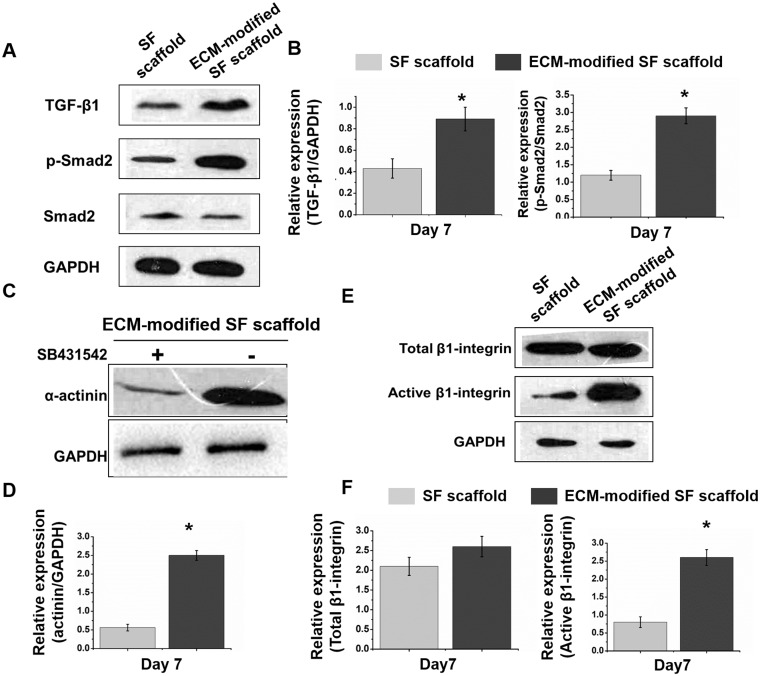
(**A**, **B**) The expression of the TGF-β1, Samd2 p-Smad2 in BASCs grown on SF scaffold and ECM-modified SF scaffold at day 7 by western blotting, and histogram showed a remarkable increase in TGF-β1and P-smad2/ smad2 in ECM-modified SF scaffold group. (**C**, **D**) The expression of α-actinin in cultured BADCs on ECM-modified scaffold after adding inhibitor (SB431542) was decreased compared to those no inhibitor at day 7. *P < 0.05. (**E**, **F**) Activated β1-integrin protein expressed in ECM-modified SF scaffolds group was 3-fold higher than SFs group at 7 day, whereas the expression of β1-integrin in these two groups shows no difference

Several signaling pathways, including transforming growth factor (TGF)-β1, bone morphogenic protein (BMP) 6 and Wnt signaling pathways, play key roles in stem cell differentiation. To further assess the potential contributions of the TGF-β1 signaling pathway, which regulates cell-derived ECM to promote cardiac differentiation in BASCs, to the activation of BASCs by cell-derived ECM, we first analyzed the secretion and protein expression of TGF-β1 by western blotting. TGF-β1 expression was increased by approximately 2-fold in BASCs on the ECM-modified SF scaffold compared with that in the control group on day 7. In addition, analysis of BASC activation by Smad phosphorylation revealed that total Smad2 expression was not altered by TGF-β1; however, Smad2 phosphorylation was significantly increased in the ECM-modified scaffold group, indicating that induction of BASC differentiation by cell-derived ECM may be attributed to TGF-β1 activation (Fig. 6A and B). To further confirm this hypothesis, we treated cells with the ALK5 kinase inhibitor SB431542 to block TGF-β1 signaling (Fig. 6C and D).

**Figure 7:**
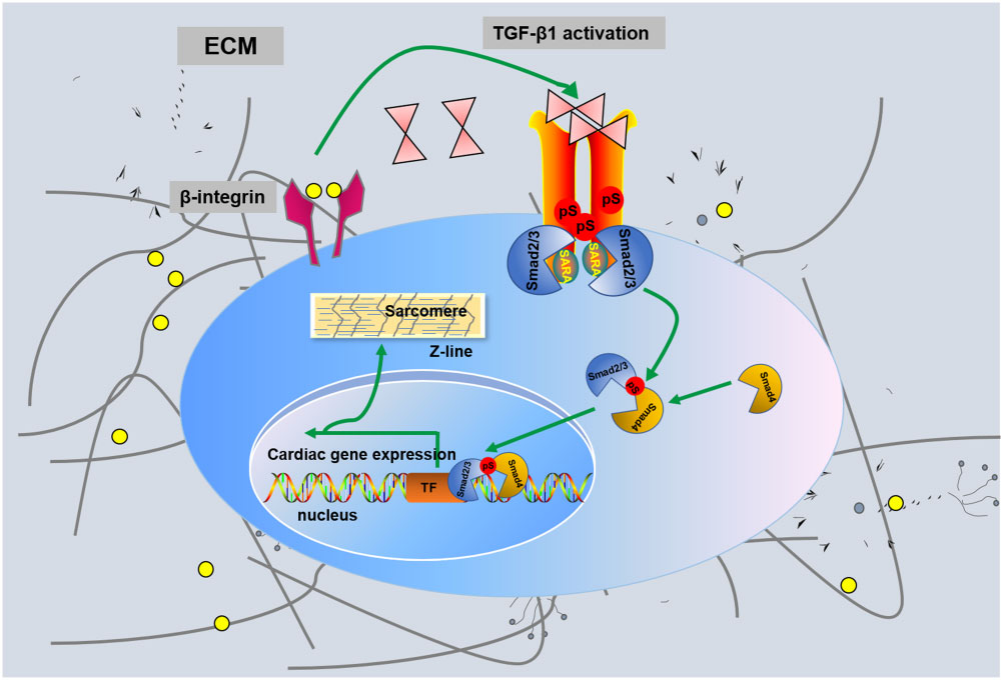
A schematic model was proposed to describe the SF-based cell-derived ECM-modified scaffold promote cardiogenesis through the β1 integrin-dependent TGF-β1 signaling pathway

Next, we further clarified the β-integrin played important role in the differentiation of BASCs into CMs owing to the cell-derived ECM through a mechanism involving the TGF-β1 pathway. First, we found that BASCs cultured on ECM-modified SF scaffolds showed approximately 3-fold higher expression of β-integrin than cells cultured on SF scaffolds, whereas β1-integrin total protein expression had no significant difference between the two groups (Fig. 6E and F). These results indicated that the β1-integrin signal was activated in BASCs by the cell-derived ECM; therefore, this mechanism may be involved in the regulation of BASC differentiation into CMs by the cell-derived ECM. Accordingly, we concluded that the β1-integrin-mediated TGF-β1 signaling pathway facilitated the differentiation of BASCs on the ECM-modified SF scaffold and speculated that TGF-β1-mediated activation of Smad2 via phosphorylation further stimulated cardiac-specific transcription factors and ultimately promoted the differentiation of BASCs into CMs (Fig. 7).

## Discussion 

Although many studies have evaluated the construction of ECTs *in vitro* based on biomimetic scaffold materials, there are still some problems associated with this approach, including insufficient biomimetic ability. Therefore, researchers have examined the potential applications of cell-derived ECM scaffolds. In this study, we developed a scaffold material with a natural myocardial ECM component and elucidated the regulation of BASCs proliferation and differentiation. Our results showed that the cell-derived ECM regulated the β1-integrin-dependent TGF-β1 signaling pathway and promoted the differentiation of BASCs into CMs.

Studies have shown that cell-derived ECM can effectively promote stem cell proliferation, adhesion and differentiation [25, 26]. Moreover, CFs are an important component of cardiac stromal cells, and the secreted ECM constitutes a myocardial interstitial network that plays a decisive role in the maintenance of normal heart structure, metabolism and function. Therefore, CF-derived ECM was obtained by continuous culture of CFs in scaffolds for 10 days and treatment with detergents, such as Triton X-100 and NH_3_. The main protein components of the ECM, including collagen I and collagen III, were confirmed by immunofluorescence staining. Collagen IV, fibronectin and laminin are also retained in cell-derived ECM materials. According to a previous report [27], CF-derived ECM may also incorporate a series of bioactive molecules that affect cell adhesion, proliferation and differentiation. Additionally, CF-derived ECM incorporates 18 biologically active factors, including TGF-β and galectin-1, which effectively regulate the biological behaviors of cells. The results also confirmed that the simple CF-derived ECM could effectively promote the differentiation of BASCs into CMs, and the differentiation efficiency was significantly improved. This result provides an important basis for the further application of CF-derived ECM-modified SF scaffolds for constructing ECTs.

SFs is a kind of natural biomaterial with physical, chemical and biological properties. These scaffolds are suitable for the construction of engineered myocardial tissue. Chi *et al.* used SF porous scaffold to carry bone marrow MSCs for rat myocardial infarction repair, confirming that the complex could effectively improve the retention and survival of transplanted cells in the myocardial infarction area [28]. However, the construction of ECTs using SF scaffolds is associated with various problems, including the presence of a single SF component, which cannot simulate a complex ECM microenvironment. In this study, CFs were cultured on SF scaffolds and then decellularized through chemical method in order to obtain cell-derived ECM-modified SF scaffold. Immunochemical staining confirmed that the modified scaffold material contained conventional ECM proteins, and this microstructure was observed by SEM. The constructed ECTs were further combined with BASCs, and the results showed that the early ECM-modified SF scaffold could effectively promote cell adhesion on scaffold materials during the construction of ECTs. DNA content determination showed that the scaffold material modified with ECM promoted the proliferation of BASCs. Additionally, we found that incorporation of ECM enhanced the maturation of BASC-derived CMs, with more sarcomere formation and increased expression of Cx43.

When using stem cells to repair damaged myocardium, further analysis of the signaling pathways that regulate the differentiation from BASCs to CMs is essential for understanding the principles of stem cell repair. Adipose tissues can be used as new sources of MSCs owing to the advantages of simple, noninvasive collection and abundance. Stem cells derived from brown fat are important sources for myocardial construction and have progressed rapidly in studies on differentiation, expansion and tissue engineering applications in recent years. Many studies have examined myocardial tissue engineering using brown adipose stem cells (BASCs) as a cell source. Transplantation of BASCs has been used for the treatment of myocardial infarction *in vivo* [29, 30]. Moreover, chitosan-based hydrogels, poly(*N*-isopropylacrylamide)/single-wall carbon nanotubes, and other materials carrying BASCs have been transplanted for repair of myocardial infarction-based damage [31]. Recent studies of BASCs have focused on improving the efficiency and stability of BASC differentiation into CMs and how to use better stents for myocardial regeneration and remodeling.

In this study, we found that CFs-derived ECM stimulates BASCs to differentiate into the CMs by activating the TGF-β1 signaling pathway. The results of Q-PCR showed that markers for early differentiation of heart, such as Mef2c, Nkx2.4, Gata4, etc., were significantly highly expressed compared with the control. It has been reported that TGF-β family members TGF-β1 and BMP stimulate the expression of cardiac-specific proteins. The physiological functions of TGF-β1 include regulation of cell adhesion, proliferation and differentiation [32–34]. Studies have shown that TGF-β1 plays an important role in cell differentiation and vascular remodeling, and induces differentiation of stem cells [33]. For example, Sun *et al.* [35] believe that TGF-β1 can induce BASCs expression and phosphorylation of Smad2 expression, and promote BASCs differentiation into CMs. This study found that phosphorylation levels of Smad2 were significantly increased accompanying with high expression of TGF-β1. Simultaneous inhibition of TGF-β1 significantly reduced the differentiation of BASCs into CMs, confirming that ECM can active TGF-β1 and then promote the development of the heart.

At the same time, we found that cell-derived ECM can promote differentiation of BASCs to CMs, the differentiation may be regulated by activating β1-integrin and triggering the TGF-β1 signaling pathway. As a key mechanical transducer, β1-integrin transmits ECM signals into cells [36, 37]. As a major sensor, β1-integrin affects the specific differentiation of stem cells into the CMs. The studies indicate that as the β1-integrin as the key mechanical transmitter, transfer physical pressure signals from the ECM into the cell. β1-integrin as the main sensor affects the specific differentiation of stem cells into the CMs [38]. Our studies indicate that cell-derived ECM activate the cell β1-integrin signaling pathway at an early stage. It is speculated that regulates the differentiation of BASCs into CMs by β1-integrin-dependent TGF-β1.

## Conclusion

In summary, we successfully developed CFs-derived ECM-coated SF scaffold and evaluated the effects of this scaffold on the differentiation of BASCs into CMs. Our findings showed that the cell-derived ECM-modified SF scaffold material contained various ECM components and significantly promoted the proliferation of BASCs and their differentiation into CMs. We also showed that CF-derived ECM increased the expression of CMs -specific proteins and stimulated the β1-integrin-dependent transforming growth factor-β1 signaling pathway.

## Supplementary data

Supplementary data are available at *REGBIO* online.

## Funding

This work was supported by the Key Program of the National Key Research and Development Program of China (nos. 2017YFA0106100, 2016YFY1101303); the National Natural Science Funds for Outstanding Young Scholar (no. 81622027); the Key Program of National Natural Science Foundation of China (no. 31830030); and the Beijing NOVA Program of China (no. 2016B615). Joint funds for National Natural Science Foundation of China (no. U1601221). 

*Conflict of interest statement*. None declared. 

## Supplementary Material

rbaa011_Supplementary_Data
