# Supplementary material for: Cell-derived extracellular matrix-coated silk fibroin scaffold for cardiogenesis of brown adipose stem cells through modulation of TGF-β pathway
Source: Regen Biomater. 2020 Apr 24;7(4):403–12. doi: 10.1093/rb/rbaa011 (PMC7415001; doi:10.1093/rb/rbaa011)
Supplement: rbaa011_Supplementary_Data [file rbaa011_supplementary_data.pdf]

Supporting Information

Supplemental Figures

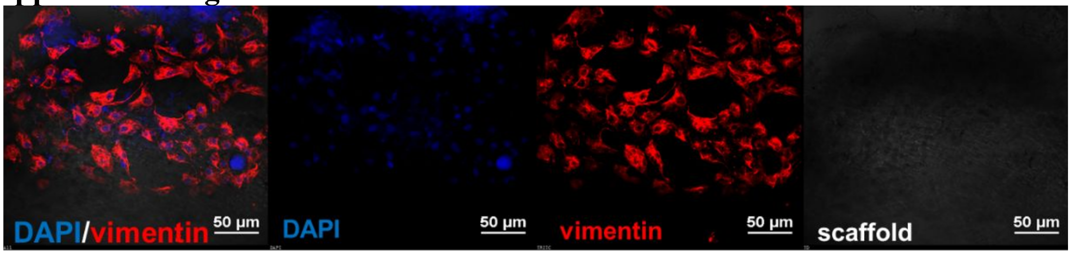

Figure S1 Immunofluorescence staining showed cardiac fibroblasts on the silk fibrin scaffold express vimentin.

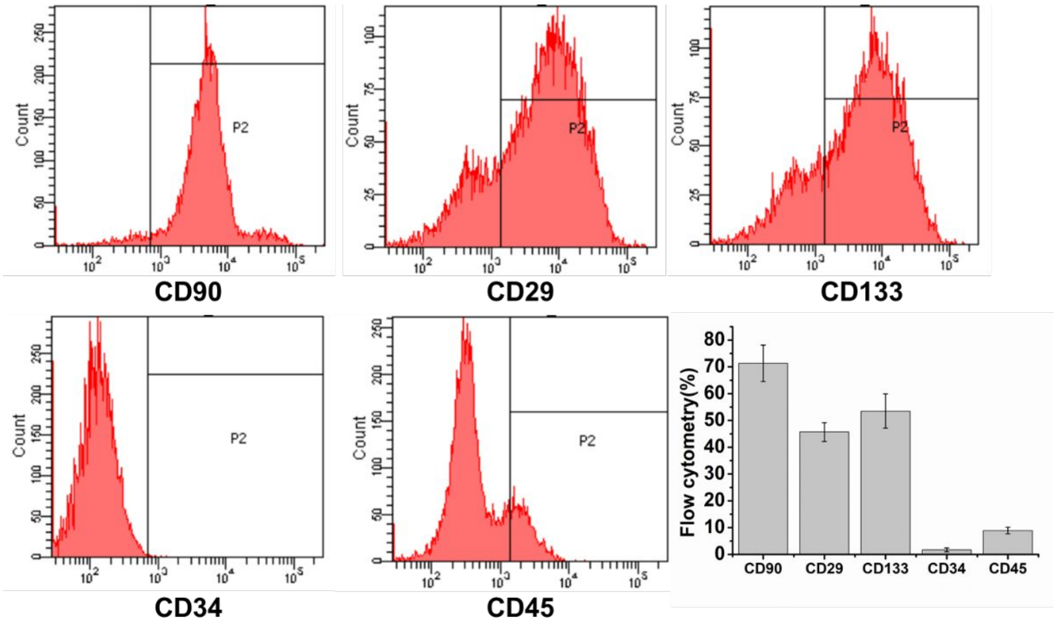

Figure S2 FACS analysis for surface markers of the isolated BASCs. These cells expressed CD90, CD29, and CD133 and but negative express CD45, a hematopoietic and leukocyte marker CD34.

Supplemental Table

Table S1: RT-qPCR primer sequences in this study.

Genes	Forward primer	Reverse primer
Mef2C	CTTTCCAGGTTGGCTCTTACTCC	GCCTCCTCCTAACAAAGTGGGTA
NKX2.5	CAAGTGCTCTCCTGCTTTC	ATCCGTCTCGGCTTTGTC
Gata4	GATGGGACAGGACACTACC	CAGTTGGCACAGGAGAGG
Isl1	CATCGAGTGTTTCCGCTGTGTAG	GTGGTCTTCTCCGGCTGCTTGTGG
α-actinin	GAACGCAAATATTCTGTCTGG	GGGCCAGCTTCGTCATAC

c-TnT	TGAACAGCAGCGTATTCG	GACAGAGCCTTCTTCTTCC
